# The effect of the mindfulness-based interventions on inflammaging: Protocol for a systematic review and meta-analysis

**DOI:** 10.1371/journal.pone.0284228

**Published:** 2023-04-07

**Authors:** Jing Lv, Haining Peng, Tengbo Yu, Xiaohong Huang

**Affiliations:** 1 Department of Psychology, School of Social Development and Public Policy, Fudan University, Shanghai, China; 2 Department of Sports Medicine, The Affiliated Hospital of Qingdao University, Qingdao, China; 3 Institute of Sports Medicine and Health, Qingdao University, Qingdao, China; 4 Department of Orthopedic Surgery, Qingdao Hospital, University of Health and Rehabilitation Sciences (Qingdao Municipal Hospital), Qingdao, China; 5 Medical Research Center, The Affiliated Hospital of Qingdao University, Qingdao, China; University of Catania: Universita degli Studi di Catania, ITALY

## Abstract

**Background:**

Inflammaging, a chronic low-grade inflammation, is considered as the basis of age-related diseases. Mindfulness is involved in protecting telomeres, whose shortening causes aging. This paper reports a protocol for the meta-analysis and systematic review to bond the causality between the mindfulness practices and inflammaging responses according to the data collected from the relevant observational studies.

**Methods and analysis:**

The published studies during 2006–2023 will be identified from PubMed, Web of Science, Cochrane Central Register of Controlled Trials, and ProQuest Dissertation & Theses Global. The retrieved records will be screened independently by two researchers, and the relevant data will be extracted after reaching an agreement. The eligible studies will be analyzed with both of a meta-analysis and a narrative review. The risk of bias will be evaluated according to the Cochrane assessment for risk of biases. In the meta-analysis, random models will be applied to evaluate the effectiveness of mindfulness-based interventions on inflammaging due to the variation among studies. The d_ppc2_ and Cohen’s d will be calculated for synthesizing the evidences from the randomized controlled trials and intervention programs without a pretest-posttest design, respectively. The interstudy heterogeneity will be assessed with the Q test and quantified using I^2^ statistic. The subgroup analyses will be conducted against the categorical moderators and meta-regressions against the continuous ones. A narrative review will be recruited to deepen the understanding of the primary outcomes, in which consequential covariates with limited data in the bulk of reports will be included.

**Trial registration:**

**PROSPERO registration number** CRD42022321766.

## Introduction

The human plasma proteome from 4,263 subjects (aged 18–95 years) profiles an undulating change in the aging-related proteins, peaking at the ages of 34, 60 and 78 [1], which is in agreement with the lifespan development theory, i.e., aging expands the entire life span [2]. The telomere length (TL) and telomerase activity (TA), decreased along with cell division, are the proposed cellular biomarkers for human aging and predictive to physical health and longevity. Longevity is a vascular question, and the onset of aging could be called as physiological arteriosclerosis [3]. Pulse wave velocity (PWV) and flow-mediated dilation (FMD) stand for the quality of arterial tissue [4,5]. Inflammaging is defined as a chronic, low-grade, controlled and asymptomatic inflammation in aging [6]. Inflammaging occurs naturally with aging, and it is accelerated by various external triggers [7]. Most if not all age-related diseases are related to an inflammatory pathogenesis [7]. Inflammaging is characterized by increased pro-inflammatory cytokines, especially interleukin-6 (IL-6), tumor necrosis factor alpha (TNF-α), and C-reactive protein (CRP). Herein, down tuning the inflammaging-associated key factors mitigates aging and prevent age-related diseases.

**Fig 1:**
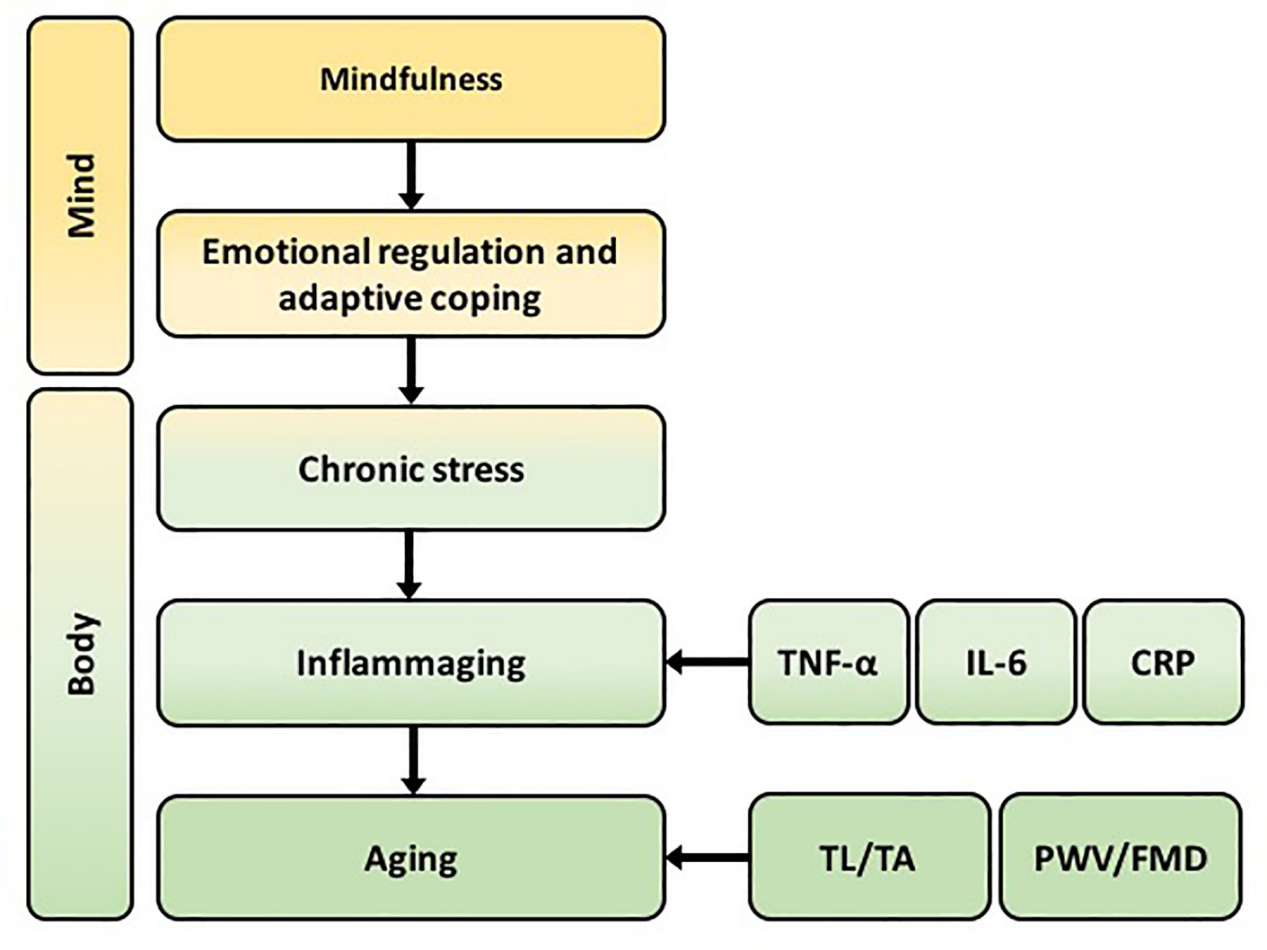
A schematic depiction of how BMIs might influence inflammaging and aging.

Mindful living maintain TL via increasing positive states of mind and body [8]. Mindfulness is a quality which benefits the psychological functioning and physical health [9]. Hence, the mindfulness practices may attenuate inflammaging, potentially promoting emotional regulation and adaptive coping and reducing chronic stress (Fig 1). Immunity-related parameters were recruited in the observational studies to evaluate the effect of the mindfulness-based interventions (MBIs), but no consensus has been reached. The only meta-analysis regarding the effect of MBIs on the immunity-related biomarkers has been reported recently, including CRP, IL-6, CD4+, TL, and TA, which quantifies MBIs’ potential in improving immune function and attenuating somatic disorders [10]. However, if mindfulness practices attenuate inflammaging and aging is still unknown. Logically, a robust meta-analysis evaluating the effectiveness of MBIs on inflammaging and aging is advocated based on all the relevant studies.

### Objectives

The protocol study is designed to investigate the effect of the MBIs on inflammaging via a systematic review and meta-analysis. The objectives of this study include:

- Illustrate the relationship between the MBIs and the biomarkers, including PWV, FMD, TL, TA, TNF-α, IL-6, and CRP;
- Explore how the types of intervention, intervention duration, control-group types, subject health condition, and participants’ age account for the outcomes of the meta-analysis.

## Methods and analysis

### Review design

The protocol has been designed based on the guide book, preferred reporting items for systematic review and meta-analysis protocols (PRISMA-P) [11]. The review method was developed in accordance with the meta-analysis of observational studies in the epidemiology: a proposal for reporting [12], PRISMA-P checklist [13], and Cochrane Collaboration Handbook [14]. The study has been registered on PROSPERO (CRD42022321766).

### Search strategy

Literature search will be conducted with Pubmed, Web of Science, Cochrane Central Register of Controlled Trials, and ProQuest Dissertation & Theses Global databases via the search terms in Table 1. Literatures will be collected until the systematic review and meta-analysis is published. The literature searching strategy combines mindfulness and nine parameters regarding to inflammaging and aging. The first theme is mindfulness, the second theme is IL-6 OR TNF-α OR CRP OR TL OR TA OR PWV OR FMD. The exploded versions of Medical Subject Heading of each theme will be included.

**Table 1:** The search strategy.

Theme				*Search terms*
***Mindfulness***	***Inflammaging***	Inflammaging	TNF-α	‘mindfulness’ AND ‘tumor necrosis factor alpha/TNF-α’
			IL-6	‘mindfulness’ AND ‘interleukin-6/IL-6’
			CRP	‘mindfulness’ AND ‘C-reactive protein/CRP’
		Aging	Telomere	‘mindfulness’ AND [‘telomere length’ OR ‘telomerase activity’]
			Arterial stiffness	‘mindfulness’ AND [‘flow-mediated dilation/FMD’ OR ‘pulse wave velocity/PWV’ OR ‘arterial stiffness’]

### Inclusion criteria

Studies related to mindfulness and mentioned about the aging and ***inflammaging*** biomarkers will be included. The inclusion criteria are 1). academic articles and dissertations published in English, 2). participants aged > 18, 3). randomized controlled design, uncontrolled pretest-posttest design, or controlled without pretest-posttest design, 4). having mindfulness in the intervention, 5). presenting at least one of the nine biomarkers in the outcomes, 6). reporting appropriate data format of the outcomes for effect size calculation. The exclusion criteria were opposite to the inclusion criteria without additional limitation.

### Data collection and management

Endnote X9 will be used in literature management. Data will be extracted and collected using the Microsoft Excel 16.3. Statistical analyses will be conducted using R 4.2.1.

### Data extraction

**Fig 2:**
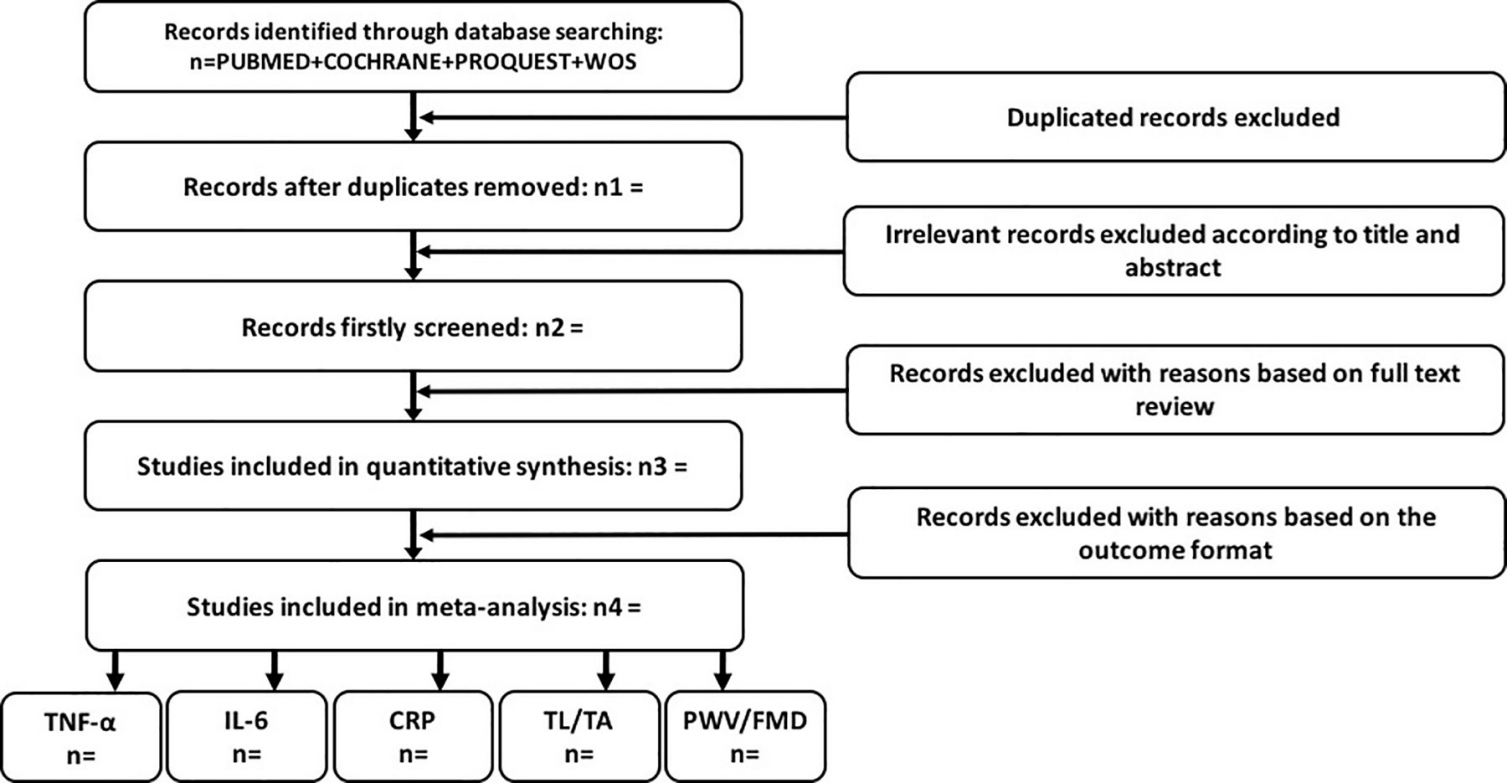
A flowchart of study selection.

For the eligibility (Fig 2), the studies retrieved from the databases will be grouped, and duplicated records will be removed. The titles and abstracts will be independently checked by two researchers following the inclusion criteria. Afterwards, the two researchers will read the full manuscripts to determine the included studies and clarify the reasons of the exclusions, independently. A third reviewer will intervene in case of disagreement, and a consensus will be sought. Two researchers will, again, independently extract the data regarding the author information, publication year of the studies, the gender, age, body mass index (BMI), health condition (e.g., healthy people, people with clinical diseases), and population (e.g., people with mindfulness practice experiences) of the participants, the type [e.g., mindfulness-based cognitive therapy (MBCT), mindfulness-based stress reduction (MBSR)] and length of intervention, study design [e.g., randomized controlled trial (RCT), uncontrolled pretest-posttest, or controlled without pretest-posttest design), and control condition (e.g., passive control, active control). A data extraction sheet will be made to include all the information mentioned. The information will be adapted in the case that relevant information is lack, and the authors of the included studies with missing key information will be contacted. The inconsistent coding will be discussed, and the final version will be confirmed among the three researchers.

### Outcomes and prioritization

The primary outcome of the meta-analysis is to record the impacts of the MBIs on PWV, FMD, TL, TA, TNF-α, IL-6, and CRP, respectively. The observational studies should report: 1) the sample sizes of the control and intervention groups (specific to pre- and post-tests for the RCTs); and 2) the mean and standard deviation values of the inflammaging or aging biomarkers for the control and intervention groups; or 3) other statistic records, such as F-test, t-test, or correlations; or 4) the effect sizes. These data will be used for synthesizing the effect size of each study and the pooled effect size further, or moderator analysis.

### Risk of bias assessment of included studies

The risk of bias for the studies included in the systematic review and meta-analysis will be evaluated according to the the Cochrane bias risk assessment tool, which is recommended by the Cochrane Handbook. The studies will be assessed from six aspects: selection bias, implementation bias, measurement bias, follow-up bias, reporting bias, and other biases [14]. Two researchers will assess the risk of bias at the study level. A third researcher will join if discrepancy occurs.

### Data synthesis

In the meta-analysis, a random effects model will be used. The standardized mean difference effect size of PWV, FMD, TL, TA, TNF-α, IL-6, and CRP from the eligible studies will be synthesized to investigate the effect of the MBIs on inflammaging [15]. To specific, the d_ppc_ [16] (standardized mean difference for a pre-post-control design) will be calculated for synthesizing the evidences from the RCTs, and Cohen’s d [17] will be calculated for synthesizing the evidences from the uncontrolled pretest-posttest and controlled without pretest-posttest designs, respectively. The heterogeneity will be tested by the Q statistic and quantified using I^2^. The trial sequential analysis (TSA) will also be used to provide more information on the precision and uncertainty of meta-analysis. Moreover, publication bias will be analyzed with funnel plots. The Trim and Fill test will be applied to suggest the need of imputing missing cases if publication bias exist [18].

Depending on the data availability, the moderators, like the intervention type, intervention duration, control-group type, subject health condition, and participants’ age, will be analyzed accounting for the heterogeneity. Subgroup analysis will be applied to the following covariates: the type of intervention, control-group type, subject health condition, and participants’ age. And, meta-regression will be applied for the continuous covariates, e.g., the intervention duration.

A systematic narrative synthesis will be conducted for the consequential covariates with only limited data in the bulk of reports, which offers a comprehensive perspective in understanding the causality between MBIs and inflammaging, according to the Grading of Recommendations Assessment, Development and Evaluation (GRADE) guidelines [19].

### Ethics and dissemination

There will be no concerns about the privacy. All the data used in the systematic review and meta-analysis are extracted from registered trials, published studies, and unpublished dissertations. No ethics approval will be needed. Results of the meta-analysis and systemic review will be disseminated by publication in a peer-reviewed journal.

## Discussion

The pace of population aging nowadays is much faster than in the past. The Decade of Healthy Aging (2021–2030) declared by the United Nations General Assembly (UNGA) seeks to reduce health inequities and improve the lives of older people [20]. There is no typical older person [2]. Some of the aged have physical and mental capacities similar to the young, while some experience significant declines in the capacities at much younger ages. Thus, one of the challenges in response to population aging is to increase the proportion of life in good health, free from age-related diseases, and maintain the physical and mental capacities.

It has been reported that the psychology-centered endeavors, including mindfulness meditation, yoga meditation, etc, diminish the impact of stressors on immune function and improve physical health [10]. Compared to the previous meta-analysis, this protocol is proposed to focus on mindfulness practices attenuating inflammaging, recruiting comprehensive biomarkers covering chronic inflammation and aging. The result of this study will help to improve life quality in the context of population aging. Moreover, mindfulness and meditation are different, though they are used together. Meditation is a general concept containing many mind training methods. It includes seated meditation, walking meditation, yoga, etc. Mindfulness is described as “the awareness that emerges through paying attention on purpose, in the present moment, and non-judgmentally to the unfolding of experience moment by moment [21]”. Meditation and mindfulness training preclude each other, but not necessarily [22]. This study rigorously sets the search terms, aiming at exploring the impact of mindfulness practices, i.e., the interventions to the mind state, on inflammaging.

The relationship between mindfulness and inflammaging will be clarified for the first time in the systemic review and meta-analysis. The effectiveness of the MBIs on the expressions of the biomarkers will be evaluated. The mechanism underlying MBIs’ affecting inflammaging will be illustrated, with the supports from the meta-analysis, moderator analyses, and narrative review. Limitation exists. We are aware that the preselection of studies meeting the inclusion criteria rarely reporting both inflammaging and aging biomarkers. Lacking correspondence of the inflammaging and aging biomarkers prevent us from determining how the relationship between inflammaging and aging is skewed by mindfulness training. Moreover, the strengths and limitations and the future directions of this study will be highlighted in processing the identified evidences. This review provides a comprehensive scope in understanding the MBIs’ role against inflammaging and aging and help to improve the human health and wellbeing by modulating emotional regulation and stress coping strategies.

## Supporting information

S1 Checklist PRISMA-P 2015 checklist. (DOCX)
